# Induction of ganglioside synthesis in *Drosophila* brain accelerates assembly of amyloid β protein

**DOI:** 10.1038/s41598-018-26294-8

**Published:** 2018-05-29

**Authors:** Yasutoyo Yamasaki, Leo Tsuda, Akemi Suzuki, Katsuhiko Yanagisawa

**Affiliations:** 1 Laboratory of Animal Models of Aging, Center for Development of Advanced Medicine for Dementia, National Center for Geriatrics and Gerontology, Morioka 7-430, Obu, Aichi 474-8511 Japan; 2 Institute of Glycoscience, Tokai University, 4-1-1 Kitakaname, Hiratsuka, Kanagawa 259-1292 Japan; 3 Center for Development of Advanced Medicine for Dementia, National Center for Geriatrics and Gerontology, Morioka 7-430, Obu, Aichi 474-8511 Japan; 4 Present Address: Institute of Molecular Biomembrane and Glycobiology, Tohoku Medical and Pharmaceutical University, 84-4-1 Komatsushima, Aobaku, Sendai, Miyagi 981-8558 Japan

## Abstract

The assembly and deposition of amyloid β protein (Aβ) is a fundamental event during the early stages of Alzheimer’s disease (AD) and cerebral amyloid angiopathy. A growing body of evidence indicates that gangliosides form a pathological platform for the generation of ganglioside-bound Aβ, which facilitates the assembly of soluble Aβs; however, the molecular mechanisms underlying the binding of Aβ to gangliosides in the brain remain unclear due to the lack of an *in vivo* system that may address this issue. In insects, including the fruit fly *Drosophila melanogaster*, gangliosides are not intrinsically present at a detectable level. We herein demonstrate that ganglioside expression is inducible in *Drosophila* via the expression of transgenes of ganglioside synthesis enzymes and the feeding of exogenous sialic acid, and also that the induction of ganglioside synthesis significantly accelerates Aβ assembly *in vivo*. Our results support the hypothesis that gangliosides are responsible for Aβ assembly *in vivo* and also provide an opportunity to develop a valuable model for basic research as well as a therapeutic strategy for AD.

## Introduction

Gangliosides are glycosphingolipids containing one or more sialic acid (SA) residues and are evolutionarily conserved among mammals. Gangliosides have been implicated in various cellular processes and functions, e.g., cellular cognition, adhesion, and differentiation^1^. Gangliosides have also been reported to be pathologically involved in the development of Alzheimer’s disease (AD), the most common type of dementia among the elderly^2^. Since the assembly and deposition of amyloid β protein (Aβ) into senile plaques is the earliest event in AD, the molecular mechanisms underlying Aβ assembly need to be elucidated in more detail. We previously identified a unique Aβ species characterized by its tight binding to the GM1 ganglioside (GM1) in the brain of an individual exhibiting the early pathological changes of AD^3^. The molecular characterization of this Aβ species, named ganglioside-bound Aβ (GAβ), prompted us to hypothesize that Aβ binds to GM1 on neuronal membranes, leading to the adoption of an altered conformation that is distinct from those of soluble and fibril Aβs, and that this altered conformation subsequently facilitates the assembly of soluble Aβs into fibrils by acting as a seed^3,4^. The GAβ hypothesis has been examined for the past two decades by our and other groups using various *in vitro* and *in vivo* systems, and the findings obtained to date strongly support this hypothesis^2^.

In the course of examining the GAβ hypothesis, an area of focus has been the region-specific deposition of variant-type Aβs that are genetically inherited. For example, the Dutch-type (E22Q) mutation causes amyloid deposition predominantly in the cerebral vessel wall^5,6^, suggesting that this wall, which is mainly composed of vascular smooth muscle cells, expresses unique gangliosides that favorably initiate the assembly of Dutch-type Aβ. In order to examine this possibility, we analyzed ganglioside species in vascular smooth muscle cells and found the exclusive expression of GM3 and, to a lesser extent, GM2^7^. Furthermore, as expected, the assembly of Dutch-type Aβ was markedly enhanced in the presence of GM3^7^.

The aim of the present study was to further examine the GAβ hypothesis using a *Drosophila* model, in which the induction of GM3 and Dutch-type Aβ was manipulated. We herein demonstrated that Dutch-type Aβ assembly was significantly accelerated in GM3-induced flies.

## Results

### Transgenic induction of GalT6 and SAT1 for LacCer production

**Figure 1:**
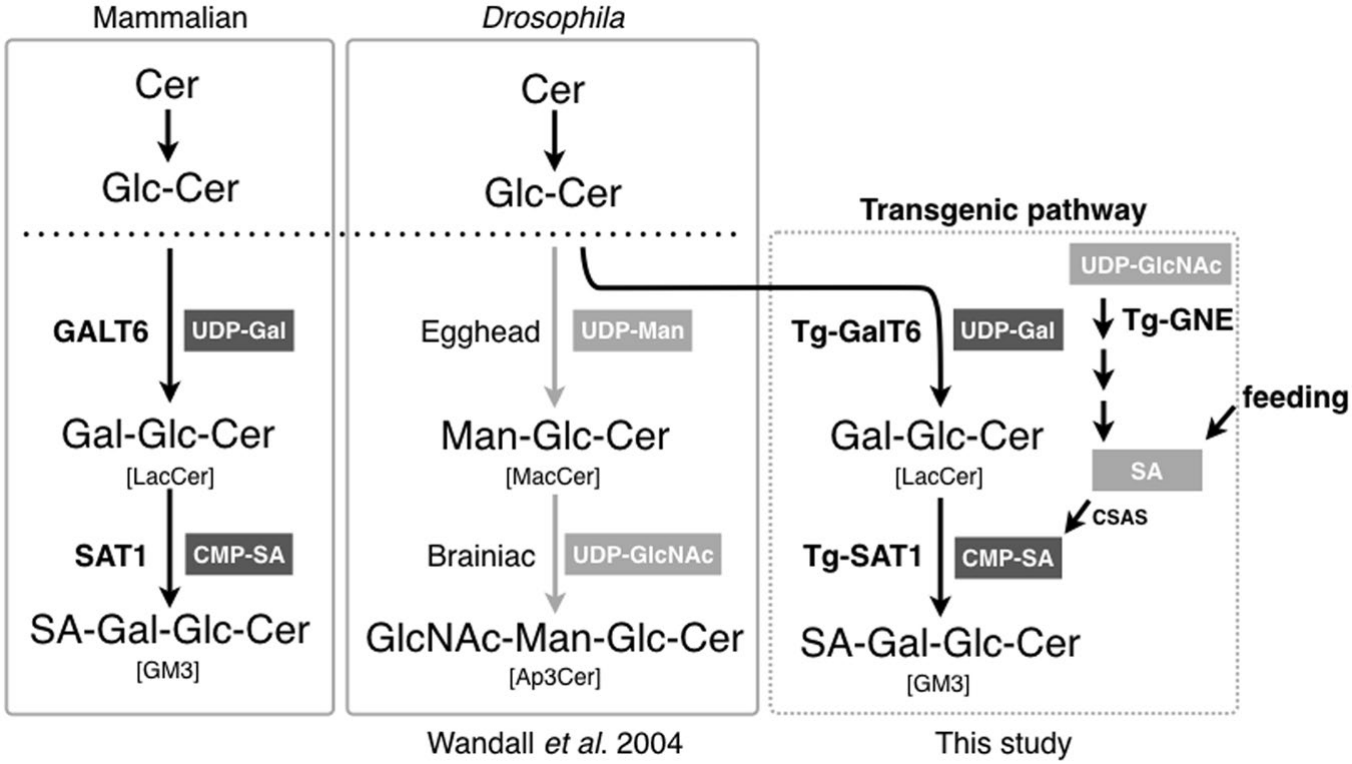
Ganglioside induction scheme. The mammalian ganglioside synthesis pathway (left), *Drosophila* endogenous pathway (middle^8^,), and *Drosophila* transgenic pathway (right, in this study). Filled boxes indicate sugar or SA donors. In vertebrates, the first ganglioside, GM3, is generated from glucosylceramide (GlcCer) via lactosylceramide (LacCer). In the *Drosophila* endogenous pathway, GlcCer is modified by the mannosyltransferase Egghead and the GlcNAc transferase Brainiac. We induced GM3 expression using transgenic (Tg) constructs and by feeding an SA donor to flies. In addition, the CMP-SA synthesis pathway is partially conserved in *Drosophila* (see Supplementary Fig. S2).

Gangliosides are glycosphingolipids and are evolutionarily conserved in mammals; however, most invertebrates, including insects, do not contain gangliosides in their cells. In the initial step in the mammalian ganglioside synthesis pathway, GM3 is generated from glucosylceramide (GlcCer) via lactosylceramide (LacCer) (Fig. 1). LacCer synthase β1,4-galactosyltransferases (GalT6/5) and the GM3 synthase α2,3-sialyltransferase (SAT1, also known as ST3GAL5 or GM3S) are responsible for GM3 synthesis (Fig. 1).

**Figure 2:**
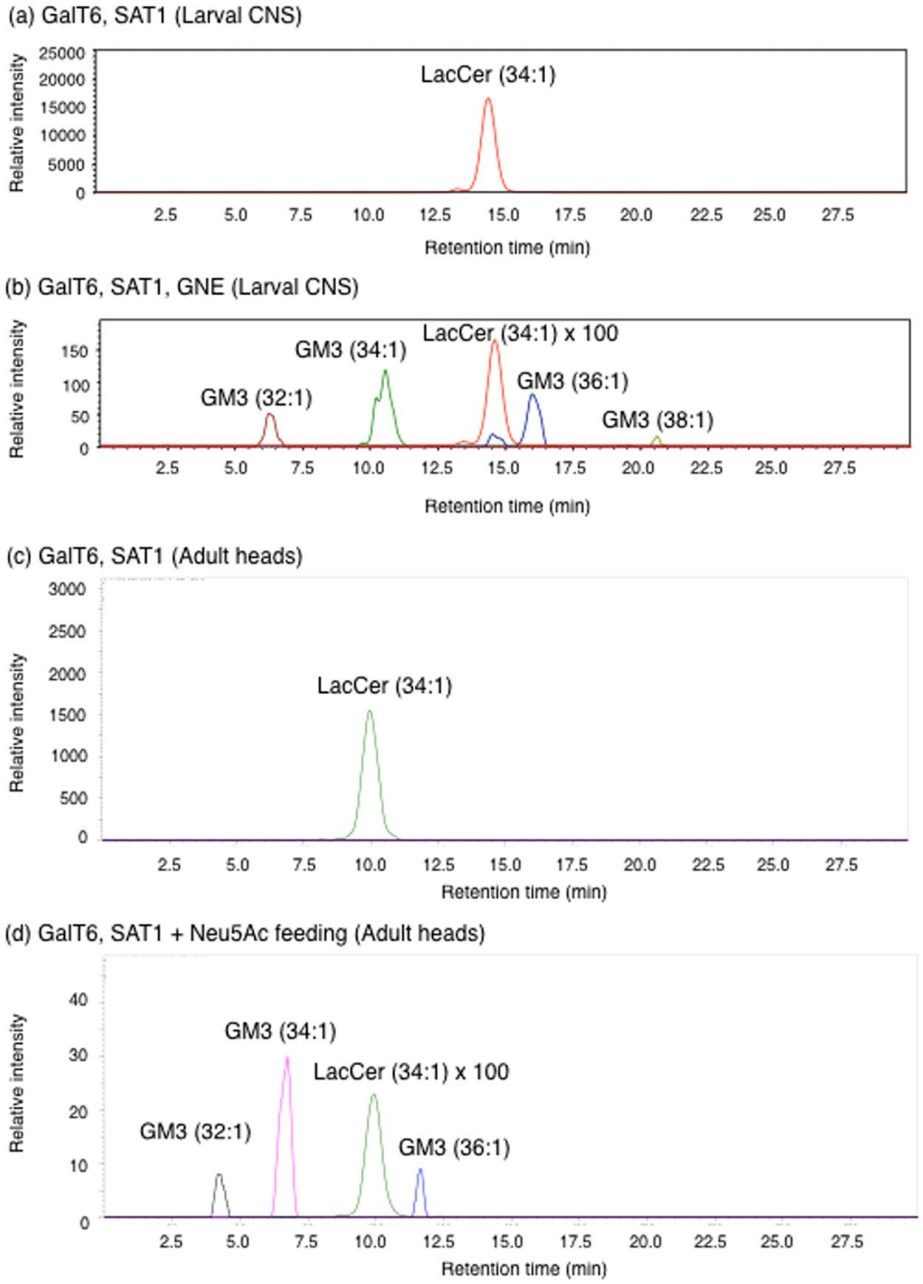
LC-MS analysis of LacCer and GM3. Extracted lipids from *Drosophila* heads were analyzed by LC-MS MRM. It was assumed that all ceramide structures of LacCer and GM3 were present based on a previous study showing that the most abundant ceramide species in *Drosophila* contained sphingosine **(**d14:1**)**^17^. Lipids from larval CNS co-expressing transgenes of (**a**) GalT6 and SAT1 or (**b**) GalT6, SAT1, and GNE. Lipids from the heads of adult flies (**c**) co-expressing transgenes for GalT6 and SAT1 or (**d**) co-expressing transgenes for GalT6 and SAT1 and fed Neu5Ac.

Since GalT6/5 and SAT1 have not been detected in *Drosophila*, we constructed a GM3 induction system with transgenic flies carrying human GalT6 and SAT1 (Fig. 1). These transgenes were expressed with the pan-neuronal driver *C155-Gal4* and total lipids were extracted from adult fly heads. By using liquid chromatography-mass spectrometry (LC-MS), we identified hexose-hexose-Cer (Hex-Hex-Cer) signals in GalT6-expressing flies, but not in non-GalT6-expressing flies (Supplementary Fig. S1). Since endogenous Man-Glc-Cer (MacCer) is a rapidly metabolized intermediate, and the transgenic GalT6 is responsible for LacCer biosynthesis, these signals are attributable to the generation of Gal-Glc-Cer (LacCer), indicating that exogenous GalT6 induces LacCer in *Drosophila*, as has been reported previously^8^ (Fig. 2a,c).

### Experimental GM3 induction in flies

We also expressed SAT1 in fruit flies with GalT6; however, GM3 was not synthesized in these flies (Fig. 2a,c and Table 1). We hypothesized that SA metabolism in insects may differ from that in mammals^9^. In support of this idea, previous studies found that the SA donor cytosine-5′-monophospho (CMP)-Neu5Ac was not detected in fruit flies, even though a functional *Drosophila* sialyltransferase has been reported^10–13^. We speculated that CMP-Neu5Ac may not be available for glycolipid modifications and proposed that this is the reason for the lack of ganglioside production in fruit flies. In order to address this issue, we employed genetic and chemical approaches.

**Table 1:** Summary of results presented in Fig. 2.

Genotype		LacCer	GM3
**a. Larval CNS**			
GalT6, SAT1		+	ND
GalT6, SAT1, GNE		+	+
**Genotype**	**Neu5Ac feeding**	**LacCer**	**GM3**
**b. Adult heads**			
Control	−	ND	ND
GalT6	−	+	ND
GalT6, SAT1	−	+	ND
GalT6, SAT1	+	+	+
SAT1	+	ND	ND

ND: not detected. (**a**) Samples from larval CNS. GalT6 and SAT1 were sufficient for LacCer production, but insufficient for GM3 production. The expression of GNE was also required to produce GM3. (**b**) Samples from adult heads. GalT6 was sufficient to produce LacCer, whereas GalT6 and SAT1 were insufficient to generate GM3. The feeding of an SA donor Neu5Ac to flies successfully induced GM3. GalT6 was required to generate GM3, suggesting that SAT1 specifically recognizes LacCer, but not MacCer.

In the genetic approach, we noted that homologs of all of the related enzymes in the SA donor synthesis pathway are present in the *Drosophila* genome, except for uridine diphosphate (UDP)-GlcNAc-2-epimerase/ManNAc kinase (GNE)^13–16^ (Supplementary Fig. S2). Thus, we hypothesized that transgenic GNE may lead to GM3 production in the background of flies expressing GalT6 and SAT1. We established a fly line carrying the human GNE gene (Supplementary Fig. S3) and extracted lipids from human GNE-expressing larval CNS samples. Using an LC-MS with multiple reaction monitoring (MRM), Neu5Ac-Hex-Hex-Cer signals were detected in these samples (Fig. 2b), suggesting that GM3 is induced in flies. However, we used a chemical approach to induce GM3 in the adult stage because the expression of human GNE resulted in lethality in transgenic flies at the late pupal stage (pharate adults). We supplied the SA Neu5Ac to adult flies expressing GalT6 and SAT1 being fed Neu5Ac *ad libitum* for more than one week. Neu5Ac-Hex-Hex-Cer signals were also detected in the heads of Neu5Ac-fed flies using LC-MS MRM analysis (Fig. 2d). Notably, in the absence of GalT6, SAT1 did not produce Neu5Ac-Gal-Glc-Cer signals (Table 1b), indicating that GM3 is induced in adult flies. We assumed all ceramide structures of LacCer and GM3 is C14-sphingenine (d14:1) based on a previous study showing that the most abundant ceramide species in *Drosophila* contained sphingosine (d14:1)^17^.

### Acceleration of Aβ assembly *in vivo* in GM3-induced flies

Previous studies demonstrated that GM3 exhibits strong affinity for Dutch-type Aβ, and the relationship between GM3 and Dutch-type Aβ accelerates *in vitro* Aβ assembly^7,18^. In order to confirm these findings using our GM3-induction system, we generated transgenic flies carrying Dutch-type Aβ40 or Aβ42 (hereafter Dut40 or Dut42, respectively). In human patients and transgenic mice with the Dutch-type mutant, Aβ40 is predominantly found in amyloid deposits^19^. We used wild-type Aβ42 (WT42) as a control for the Aβ-production pathway because sporadic-type AD patients and Aβ precursor protein-transgenic mice show Aβ42-dominant deposits, and wild-type Aβ has markedly weaker affinity than Dut40 for GM3^18^.

**Figure 3:**
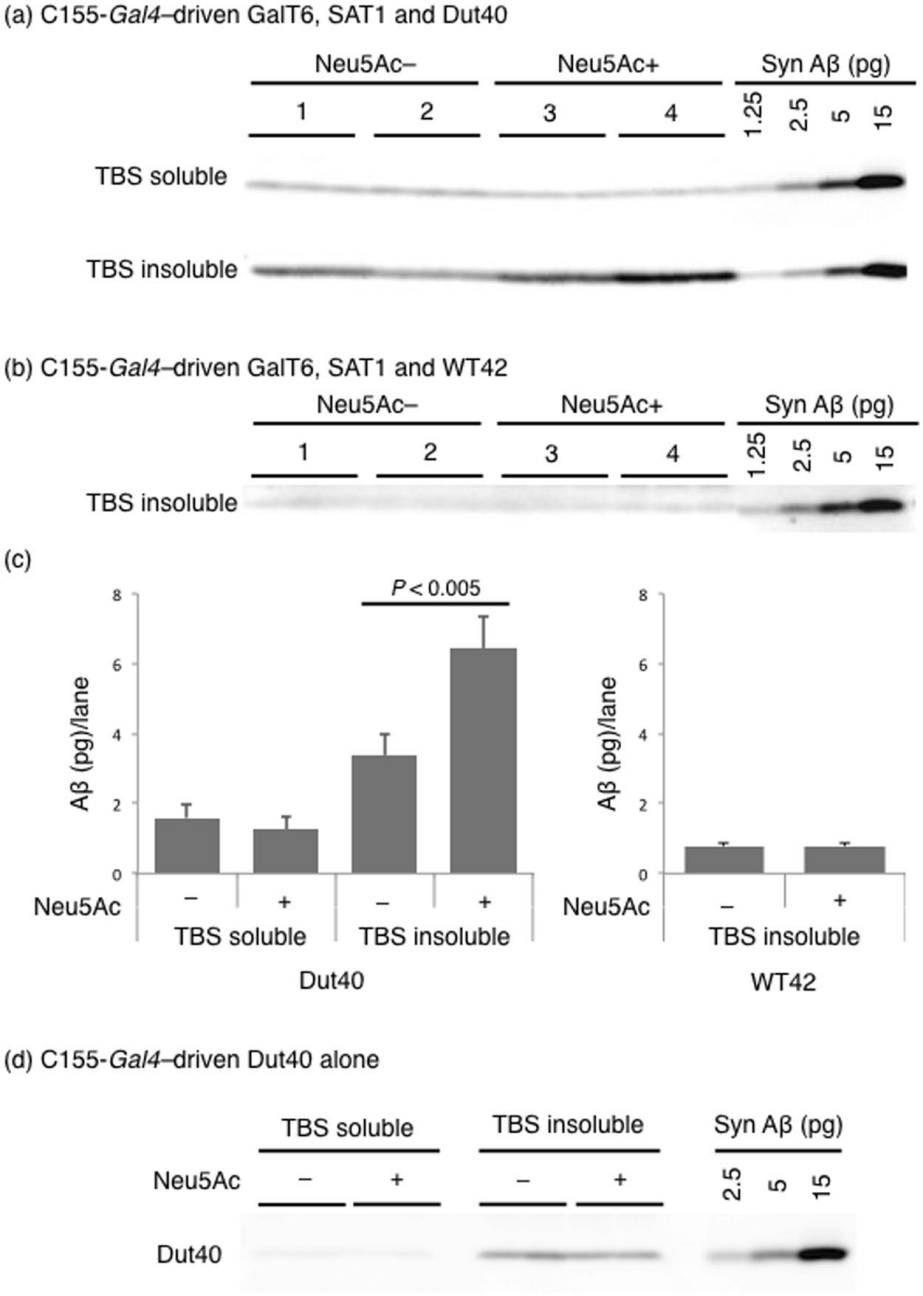
Analysis of Aβ assembly by Western blotting. Whole extracts of the heads of 10 flies from each group were separated into TBS-soluble and -insoluble fractions. The latter fraction included assembled Aβs. Syn Aβs were used as a quantitative control (Supplementary Fig. S4). The full-length blots are included in a Supplementary Figure S5. (**a**,**b**) Western blotting of Aβ from GalT6- and SAT1-expressing flies. Two independent samples from each of the two fractions (1–4) were prepared and each was loaded onto three adjacent lanes. GM3 enhances the assembly of Dut40 (**a**), but not WT42 (**b**). (**c**) Quantification of a and b. Error bars indicate the standard error of the means. A significant difference was observed in the amount of Aβ in the TBS-insoluble fraction between GM3+ (Neu5Ac+) and GM3− (Neu5Ac−) flies, but not in the amount of Aβ in the TBS-soluble fraction (n = 3, *P* < 0.005, Student’s *t*-test). (**d**) Neu5Ac feeding alone did not enhance Aβ assembly in flies without GalT6 and SAT1 transgenes, suggesting that GM3 is required.

Transgenic GalT6, SAT1, and WT42 or Dut40 were continually expressed in the fly nervous system, and GM3 was induced in these flies by the Neu5Ac feeding as described above. Whole extracts of adult fly heads were separated into TBS-soluble and -insoluble fractions. The synthesis of GM3 accelerated the assembly of Dut40 in their brains (Fig. 3a,c). Conversely, WT42 did not accelerate assembly under these conditions (Fig. 3b,c). The feeding of Neu5Ac only did not enhance Dut40 assembly in non-GalT6/SAT1-expressing flies (Fig. 3d), suggesting that Dut40 is more prone to assemble in the presence of GM3 *in vivo* and that GM3 at these levels does not affect Aβ production.

## Discussion

In the present study, we developed the first ganglioside-induction system in *Drosophila* through transgenic and chemical approaches. Although the limited amount of induced gangliosides did not allow us to perform a complete characterization of GM3 structure via an MS^n^ and NMR analyses, we concluded that GM3 was successfully generated in *Drosophila* based on the following results: Neu5Ac was required for Neu5Ac-Hex-Hex-Cer production, and Neu5Ac-Hex-Hex-Cer signals were dependent on the existence of GalT6, indicating that SAT1 specifically recognizes induced LacCer, but not endogenous MacCer (Table 1). Using this system, we revealed that GM3 induction resulted in the accelerated assembly of Aβ in *Drosophila*.

We propose three explanations for poor GM3 production in fly cells. The SA-delivery system may not be optimal for the proper induction of GM3; although we confirmed the intake of Neu5Ac in the gut, the proportion of the Neu5Ac supplied that was delivered across the blood-brain barrier currently remains unclear. Furthermore, GM3 may be subject to enzymatic digestion in flies, even though we did not find a homolog of Neu3 sialidase, a GM3 degrading enzyme, in a BLAST search of the *Drosophila* genome. Moreover, another enzyme(s) or factor(s) may be required in addition to SAT1 and GNE for the robust production of GM3. We intend to clarify these points in future studies.

GM3, the ganglioside with the simplest chemical formula, plays important roles in multiple cellular processes, e.g., immune responses, cell migration, and the establishment of the proper structure of mammalian sensory cells^20–22^. A recent study suggested that the nematode worm *Caenorhabditis elegans* produces several different gangliosides^23^; however, difficulties have been associated with clarifying the biological significance of specific gangliosides. Since *Drosophila* does not express any type of ganglioside, our induction system for gangliosides in *Drosophila* may be used to assess the function(s) of a specific ganglioside(s) in various biological processes. Mammalian and insect cells are both known to synthesize GlcCer from ceramide for the production of complex glycosphingolipid species; however, insects convert GlcCer into MacCer instead of LacCer (Fig. 1). Consistent with previous findings, the present results suggest that GalT6 promotes the production of LacCer in *Drosophila*^8^.

To the best of our knowledge, this is the first study to confirm the accelerated assembly of Dut40 under the condition of GM3 induction *in vivo* and has three implications for AD research. This study adds additional support to our GAβ hypothesis^3,4^, indicating that the assembly and deposition of soluble Aβs is a controlled process that requires a specific, favorable environment. Taken together with the previous finding that amyloid fibrils formed in the presence of gangliosides are more toxic than those formed in solution^24^, our system may be useful for screening and then developing further efficient and suitable small compounds or antibodies to inhibit amyloidogenesis in the human brain. In this context, it is of note that GM3-bound Dutch-type Aβ has the same structure as GM1-bound wild-type Aβ, which is generated in the brains of sporadic-type AD^18^. In this system, GM3 appears to function as a scaffold Aβ assembly, and, thus, the expression levels of Aβ may be suppressed to a minimum level, thereby avoiding the artificial effects of overexpressing amyloid precursor proteins^25,26^. Our *Drosophila* GM3 induction system provides a powerful new genetic tool that will contribute to our understanding of the relationship between Aβ and ganglioside *in vivo*. In future studies, by expressing transgenes in specific cells, such as mushroom body neurons, the insect counterpart of the mammalian hippocampus, which is the brain region for memory formation^27^, will also allow us to assess Aβ toxicity using behavioral experiments.

Gangliosides, in addition to being involved in the pathological process of AD development, are also a factor in other human diseases, e.g., type 2 diabetes mellitus, deafness, tumor invasion, and Parkinson’s disease (PD)^22,28–30^. In type 2 diabetes mellitus patients, elevated GM3 levels cause insulin-receptor inactivation through a disorder in membrane microdomains, leading to insulin resistance^28^. Conversely, the loss of GM3 induces an auditory dysfunction because it is required for maintaining the proper structure of cochlear hair cells^22^. A reduction in GM3 induction was previously shown to be associated with malignant transformation^29^. In addition, a recent study reported that an *in vitro* ganglioside treatment resulted in the accelerated assembly of α-Synuclein, a major causative factor for PD^31^. Thus, our inducible GM3 induction system in *Drosophila* may contribute to our understanding of the mechanisms underlying the development of these human diseases.

## Methods

### Fly stocks

Fly stocks were maintained on standard cornmeal-yeast agar medium at 25 °C under a 12-h light/dark cycle. The *elav-GAL4*^*c155*^ (*C155-Gal4*) line was obtained from the Bloomington Drosophila Stock Center (Indiana University). The *w*^1118^ strain served as the wild type. *UAS-sec:A*β_*1-42*_^*wild type*^ (*UAS-WT42*) was generated in our previous study^32^. *UAS-GalT6*, *UAS-SAT1*, *UAS-GNE*, and *UAS-sec:A*β_*1-40*_^*E22Q*^ (*UAS-Dut40*) were generated in the present study (described below).

### Construction of transgenes

Construction of the Aβ transgene was previously described^32^. Briefly, the Aβ sequence was PCR-amplified from HeLa cell cDNA and fused to the rat proenkephalin-A precursor signal peptide^33,34^. The Dutch-type Aβ mutation (underscored) was introduced into the cloned Aβ sequence by PCR amplification (*Pfu* DNA polymerase, Promega) in conjunction with the primers: 5′ GTGTTCTTTGCACAAGATGTGGGTTCAA 3′ (forward) and 5′ TTGAACCCACATCTTGTGCAAAGAACAC 3′ (reverse), after which the product was subcloned into a pUAST vector^35^ with *Bgl*II sites.

The coding sequences of human *GalT6*, *SAT1*, and *GNE* were amplified from human brain cDNA (Takara), cloned into a pCR-Blunt II-TOPO vector (Life Technologies), and then individually subcloned into *EcoR*I sites of a pUAST vector. The following primers were used. *GalT6* cloning: 5′ ATGTCTGTGCTCAGGCGGAT 3′ (forward) and 5′ CTTGCCACGACAGCCACTTC 3′ (reverse); *SAT1* cloning: 5′ CATTAGTATGCGGACGAAGG 3′ (forward) and 5′ GCTCTCAGAGTTAGAGTTGC 3′ (reverse); *GNE* cloning: 5′ CATGGAGAAGAATGGAAATAACCGA 3′ (forward) and 5′ CTAGTAGATCCTGCGTGTTG 3′ (reverse). Each transgene was injected into *yellow white* embryos then outcrossed with the *w*^1118^ strain.

### GM3 induction and mass spectrometry

Adult flies were given *ad libitum* access to filter paper soaked with 10 mM *N*-acetylneuraminic acid (Neu5Ac, Sigma) in 150 mM sucrose every other day for one week. We observed Neu5Ac being taken up by flies by adding food dye to the Neu5Ac solution (data not shown). Lipid extraction was performed as described previously^36^. Between 200 to 1000 larval brain-disc complexes and adult fly heads were dissected from flies, each set of organs was individually pooled and homogenized in TBS (50 mM Tris-HCl, pH 7.6, 150 mM NaCl), and the homogenates were then evaporated using a centrifugal concentrator (Tomy). Pellets were suspended in chloroform:methanol 2:1 (v/v) and centrifuged at 17,800 × *g* at 20 °C for 2 min. The two solvent extracts were combined and dried at 42 °C under N_2_ gas.

The extracted lipids from each pellet were treated with 1.9 mL methanol and 0.1 mL 2 N NaOH at 40 °C for 2 h, neutralized with 0.1 mL of 2 N acetic acid, evaporated to dryness under N_2_ gas, and then desalted individually through a C18 BondElut 300 mg cartridge. Glycosphingolipids were recovered in methanol and analyzed by liquid chromatography-mass spectrometry (LC-MS). The prepared samples were dissolved in methanol and injected aliquots were separated by LC using a Develosil C30 column (1 mm i.d. × 50 mm; Nomura Chemical Co) and programmed elution solvent system composed of A: 25% aqueous ammonia/acetic acid/water/methanol/isopropanol (0.1:0.1:25:25:50 for larval brain-disc complexes or 0.1:0.1:20:30:50 for heads, v/v) and solvent B: 25% aqueous ammonia/acetic acid/water/methanol/isopropanol (0.1:0.1:2:48:50, v/v) under the gradient condition (0% B in A for 5 min, 0–100% B in A for 20 min, and 100% B for 10 min) at a flow rate of 50 μl/min. Separated glycosphingolipids were detected by Shimadzu LC-MS 8050 operating in the multiple reaction-monitoring (MRM)/negative-ionization mode. Heat block, interface, and desolvation line temperatures were 400 °C, 300 °C, and 250 °C, respectively. Nebulizer, heating, and drying gas flows were 3 L/min, 10 L/min, and 10 L/min, respectively. The monitored precursor and product ions were 1123.6 and 290.1 for GM3(32:1); 1151.7 and 290.1 for GM3(34:1); 1179.7 and 290.1 for GM3(36:1); 1207.7 and 290.1 for GM3(38:1); and 860.6 and 536.5 for lactosylceramide (LacCer; 34:1). The collision energy was 50 eV for GM3 and LacCer.

### Western blots

The heads of 10 flies carrying a gene from each transgenic line were homogenized in 100 μL cold TBS containing protease inhibitor cocktail (Roche). Homogenates were sonicated by BioRuptor (CosmoBio) and then centrifuged at 100,000 × *g* at 4 °C for 1 h (Optima Max-TL, Beckman) after which the supernatants were collected as TBS-soluble fractions. TBS-insoluble pellets were homogenized in 100% formic acid and centrifuged at 17,800 × *g* at 25 °C for 20 min. The supernatants were collected, and formic acid was evaporated by the centrifugal concentrator. Each fraction was electrophoresed through a sodium dodecyl sulfate-polyacrylamide gel (4–20% Tris-tricine gel, Cosmobio). A dilution series of commercially supplied synthetic wild-type Aβ42 (Syn Aβ) (Peptide Institute) was simultaneously loaded as a quantitative control. The gel was subsequently electrotransferred onto a nitrocellulose membrane (NitroBind, MSI). The membranes were incubated in PBS (pH 7.4, Takara) at 95 °C for 5 min, blocked in 5% (w/v) skim milk in PBS containing 0.1% (v/v) Tween 20, and then blotted with an 82E1 monoclonal anti-Aβ antibody (IBL). Goat anti-mouse HRP was used as a secondary antibody. The chemiluminescent intensity of the ECL detection reagent (GE Healthcare Life Sciences) was quantified using ImageJ (NIH).

## Electronic supplementary material

Supplementary Information
