# Supplementary material for: Induction of ganglioside synthesis in Drosophila brain accelerates assembly of amyloid β protein
Source: Sci Rep. 2018 May 29;8:8345. doi: 10.1038/s41598-018-26294-8 (PMC5974419; doi:10.1038/s41598-018-26294-8)
Supplement: Supplementary file 1 — Supplementary Information [file 41598_2018_26294_MOESM1_ESM.docx]

**Supplementary information**

Induction of ganglioside synthesis in *Drosophila* brain accelerates assembly of amyloid β protein

Yasutoyo Yamasaki^1^, Leo Tsuda^1^*, Akemi Suzuki^2†^ & Katsuhiko Yanagisawa^3^

^1^Laboratory of Animal Model of Aging, Center for Development of Advanced Medicine for Dementia, National Center for Geriatrics and Gerontology, Morioka 7-430, Obu, Aichi 474-8511, Japan

^2^Institute of Glycoscience, Tokai University, 4-1-1 Kitakaname, Hiratsuka, Kanagawa 259-1292, Japan

^3^Center for Development of Advanced Medicine for Dementia, National Center for Geriatrics and Gerontology, Morioka 7-430, Obu, Aichi 474-8511, Japan

*Correspondence to LT

Tel.: +81-562-46-2311 (ex. 7523)

Fax: +81-562-44-6594

E-mail: ltsuda@ncgg.go.jp

^†^Present address: Institute of Molecular Biomembrane and Glycobiology, Tohoku Medical and Pharmaceutical University, 84-4-1 Komatsushima, Aobaku, Sendai, Miyagi 981-8558, Japan

**
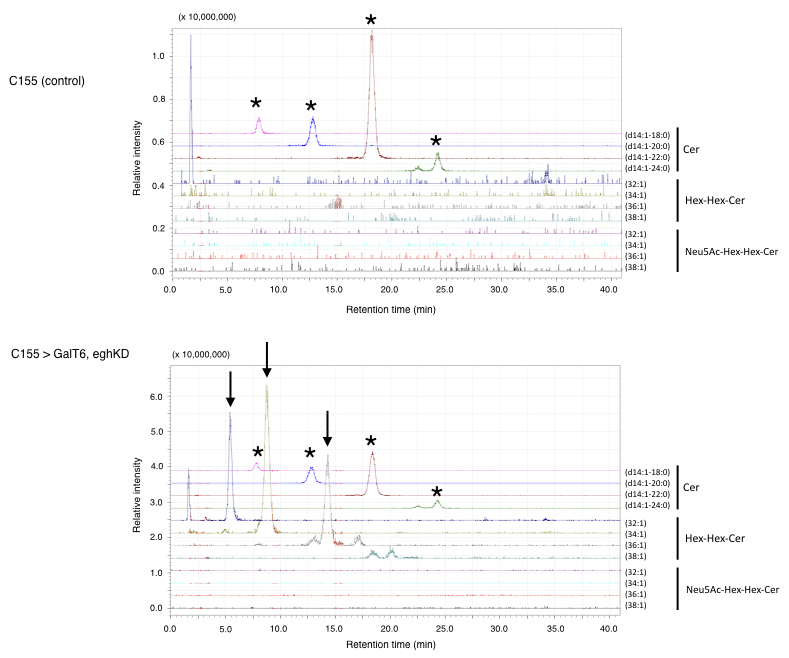
Supplementary Figure S1**

LC-MS analysis of Cer (asterisks) and LacCer (arrows) from *Drosophila* heads. In control flies, no Hex-Hex-Cer signals were detected (top). Transgenic GalT6 and the knockdown of *Drosophila* mannosyltransferase *egghead* (eghKD) induced Hex-Hex-Cer (bottom). The LC-MS analysis was performed on a Shimadzu LC-IT-TOF instrument using a Develosil C30 column (1-mm i.d. × 50 mm; Nomura Chemical Co) and the programmed elution of solvent A: 25% aqueous ammonia/acetic acid/water/methanol/isopropanol (0.1:0.1:20:30:50, v/v) and solvent B: 25% ammonia/acetic acid/water/methanol/isopropanol (0.1:0.1:2:48:50, v/v) in a gradient (0% B for 5 min, from 0 to 100% B for 30 min, and 100% B for 5 min) at a flow rate of 50 μL/min. Ceramides and glycosphingolipids were analyzed by MS in the negative ion mode and automatic scanning from *m*/*z* 200 to 2000 for MS and for tandem MS (MS^2^). Ceramides indicated by stars were characterized by MS^2^. Hex-Hex-Cer indicated by arrows were characterized by MS^2^. It was not possible to characterize the ceramide structures of Hex-Hex-Cer by MS^3^.

**
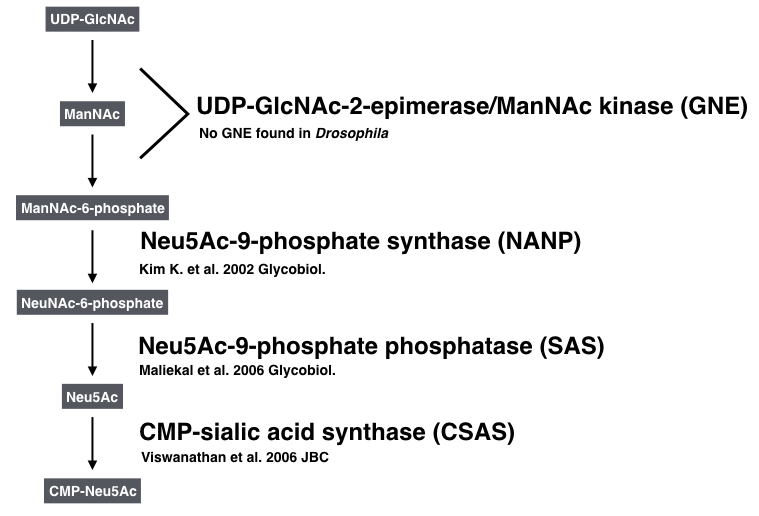
Supplementary Figure S2**

The SA donor CMP-Neu5Ac synthesis pathway in mammals and partially conserved enzymes in *Drosophila*.

**Supplementary Figure S3**

The expression of GalT6, SAT1, and GNE was assessed by immunohistochemistry. The C155–*Gal4*-driver induces UAS-transgenes in neurons (top). The induced transgenes were detected in the neuronal area (dotted lines) in the eye discs of third instar larvae. These antibodies were not reacted in the non-transgenic larvae (bottom). Anterior to the left. The following rabbit polyclonal antibodies were used: anti-GalT6 (20148-1-AP, Proteintech), anti-SAT1 (ab107534, Abcam), and anti-GNE (25079-1-AP, Proteintech). Immunostaining was performed as previously described [Yamasaki *et al.* Genes Cells. 2011].

**
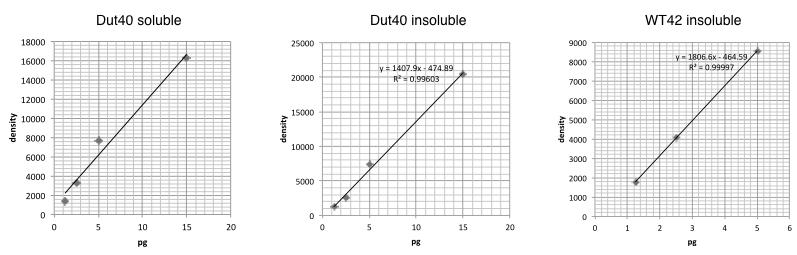
Supplementary Figure S4**

Standard curves of Syn Aβ for the quantitation of Aβ in Western blots of Figure 3 (see Methods).


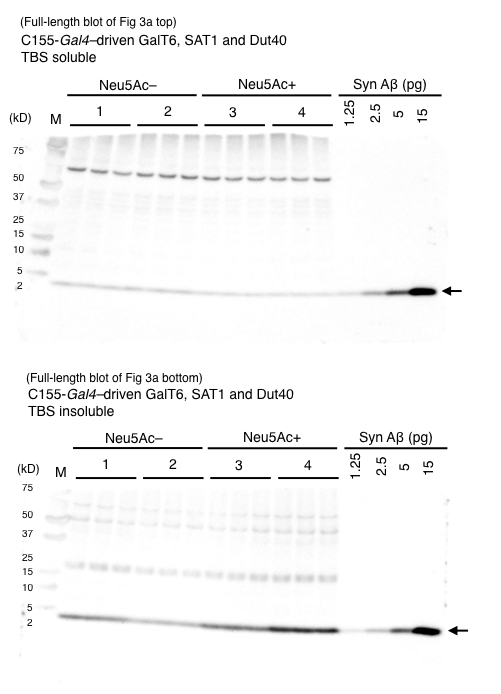

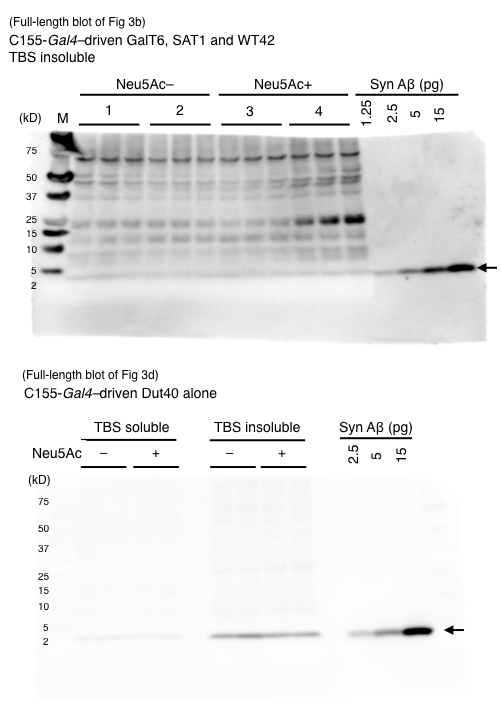


**Supplementary Figure S5**

Full-length blots of fig. 3. Arrows indicate Aβ.
